# A laboratory-based study examining the properties of silk fabric to evaluate its potential as a protective barrier for personal protective equipment and as a functional material for face coverings during the COVID-19 pandemic

**DOI:** 10.1371/journal.pone.0239531

**Published:** 2020-09-18

**Authors:** Adam F. Parlin, Samuel M. Stratton, Theresa M. Culley, Patrick A. Guerra

**Affiliations:** Department of Biological Sciences, University of Cincinnati, Cincinnati, Ohio, United States of America; Thomas Jefferson University, UNITED STATES

## Abstract

The worldwide shortage of single-use N95 respirators and surgical masks due to the COVID-19 pandemic has forced many health care personnel to use their existing equipment for as long as possible. In many cases, workers cover respirators with available masks in an attempt to extend their effectiveness against the virus. Due to low mask supplies, many people instead are using face coverings improvised from common fabrics. Our goal was to determine what fabrics would be most effective in both practices. Under laboratory conditions, we examined the hydrophobicity of fabrics (cotton, polyester, silk), as measured by their resistance to the penetration of small and aerosolized water droplets, an important transmission avenue for the virus causing COVID-19. We also examined the breathability of these fabrics and their ability to maintain hydrophobicity despite undergoing repeated cleaning. Laboratory-based tests were conducted when fabrics were fashioned as an overlaying barrier for respirators and when constructed as face coverings. When used as material in these two situations, silk was more effective at impeding the penetration and absorption of droplets due to its greater hydrophobicity relative to other tested fabrics. We found that silk face coverings repelled droplets in spray tests as well as disposable single-use surgical masks, and silk face coverings have the added advantage over masks such that they can be sterilized for immediate reuse. We show that silk is a hydrophobic barrier to droplets, can be more breathable than other fabrics that trap humidity, and are re-useable via cleaning. We suggest that silk can serve as an effective material for making hydrophobic barriers that protect respirators, and silk can now be tested under clinical conditions to verify its efficacy for this function. Although respirators are still the most appropriate form of protection, silk face coverings possess properties that make them capable of repelling droplets.

## Introduction

Personal protective equipment (PPE), specifically N95 respirators and surgical masks, are vital to protect against viral transmission during the current COVID-19 pandemic, yet global shortages of these items will likely continue in many locations for the foreseeable future. Although respirators and masks used by health care providers (HCP) and essential workers (EW) form part of the critical armament against COVID-19, a significant drawback of PPE are that they are purposed for only single use. Sterilization of PPE, especially respirators, has been implemented to enable their continued and repeated use, but this approach reduces the ability of respirators to effectively block particles, can induce damage, or may render the equipment unsafe for further usage [1].

In some cases, HCPs and EWs may only have a single respirator provided to them at their workplace and must reuse them indefinitely under hazardous work conditions. To prolong the life of respirators, many HCPs have adopted the clinical practice of wearing multiple pieces of PPE simultaneously, e.g., a mask on top of a respirator [2–4]. Clinically, this strategy is unsustainable as layering masks over respirators can negatively impact the wearer psychologically (e.g., increased and prolonged thermal discomfort while working due to the extra layer) and physiologically (e.g., further strain on breathing due to increased thickness) [2–4]. The additional mask layer also increases moisture near the wearer’s face, thus becoming a conduit for viral transmission [5, 6]. During the COVID-19 pandemic, the use of surgical masks as an additional layer is also problematic, as masks cannot be adequately cleaned without compromising their protective properties [1]. The use of masks for this task can also be logistically difficult because masks are relatively costly due to current high demand and are in short supply in their own right. In many cases, HCPs and EWs remain vulnerable as they have resorted to using (and reusing) less efficient masks on their own when respirators are unavailable, leaving them at greater risk to viral transmission.

PPE shortages are now affecting the general population, especially employees instructed to wear masks in the workplace as well as people in public places where mask wearing is mandatory or strongly recommended as part of public health policy [7, 8]. As a result, the majority of the general public has been reduced to using improvised face coverings constructed from commercially available materials. Although the primary purpose of face coverings is to minimize potential viral transmission from the wearer to others [9, 10], they can also provide some protection to the wearer from external sources [11, 12].

To help combat the PPE shortage for HCPs and EWs amid the COVID-19 pandemic, our first objective was to examine what commonly available materials can serve as immediate solutions for fashioning effective protective layers that can increase the longevity of respirators and the effectiveness of masks, such as when used under clinical conditions. For example, the Centers for Disease Control and Prevention (CDC) recommends that respirators be discarded when they have become wet, visibly dirty, or contaminated with human bodily fluid secretions [13]. Currently, there is much discussion as to how to immediately protect respirators during the COVID-19 pandemic [14], in particular using commonly available materials, but available information on the topic remains limited to anecdotal observations [14]. We therefore conducted a laboratory study to examine commonly available materials, i.e., cotton, polyester, and silk, for their suitability as a protective layer for respirators. An important feature of a suitable material would consist of its ability to protect the respirator from becoming wet and contaminated from fluids due to droplets, in a similar manner as surgical masks. Moreover, we tested materials to also see if they would not further exacerbate breathability problems associated with wearing layered PPE [2–4] and that they could be cleaned for repeated use yet retain their function. Our second objective was to examine which of these same commonly available materials would be beneficial for the construction of face coverings to be worn according to current public health guidelines when standard PPE is not available. Currently, different materials, spanning from natural to synthetic fabrics [12, 15–17], are being used for constructing both commercially sold or do-it-yourself face coverings. It remains, however, an open question as to what material possesses the best suite of characteristics to block droplets and viral particles, as well as what material best facilitates comfort, wearability, and reuse of face coverings.

In our current study, we conducted laboratory tests that examined and compared different commonly available materials, i.e., cotton, polyester, and silk, in their level of hydrophobicity, for their use as either a protective layer for respirators or as a material for constructing face coverings. Hydrophobicity is a measure of the ability of a material to repel small liquid droplets, thereby preventing the penetration and absorption of droplets, which are a vehicle for the transmission of the virus that causes COVID-19 [5]. In addition, we also compared these materials as to their breathability and to their functionality after cleaning for reuse. These two additional properties are particularly advantageous for face coverings and can facilitate their use by the general public.

Cotton is a ubiquitous natural plant-based fabric and has been employed as a useful material for face coverings during previous pandemics [15–18], most notably during the flu pandemic of 1918–19 [19] and during both the more recent SARS and H1N1 respiratory outbreaks [5, 9, 11, 12]. Previous work using aerosol particulate tests has shown that cotton fabric can provide a level of protection to the wearer due to its filtration efficiency, in particular cotton fabrics with tight weaves and low porosity [12]. A potential drawback of cotton, e.g., for use as protective layers for respirators and as material for face coverings, is that because cotton fibers are made of cellulose, it is a hydrophilic material that readily absorbs liquid [20]. Cotton’s hydrophilicity is further amplified since it can also absorb liquid via capillary action [21]. Such hydrophilicity may cause cotton to continually collect and trap droplets when used as a protective layer for respirators or when worn as a face covering, creating over time a potentially dangerous reservoir of viral particles in direct contact either with the respirator or the face of the wearer, depending on use.

Materials like polyester are petroleum-based synthetic fabrics that are currently used for different forms of PPE, e.g., barrier coats, and can vary in their levels of hydrophobicity. As a resource material, artificial fabrics like polyester can therefore offer a level of splash protection, and to further their usefulness, can be subjected to nano-treatment to improve their water repellency and to convey antimicrobial properties [5]. A potential limitation of polyester as a material, however, is that it is not a breathable fabric. Being non-breathable, polyester appears to be a suboptimal material for layering over respirators as it can further exacerbate breathability issues while wearing PPE. When worn around the face as a face covering, polyester can increase local humidity, which can create a conduit for viral transmission [5, 6]. In particular, for people in humid environments, polyester fabric can cause discomfort that can lead to manipulation or premature removal of face coverings, thereby increasing the potential for coming into contact with viral particles. Moreover, as a synthetic, pure polyester can be an irritant to people with sensitive skin and is therefore blended with other fabrics, e.g., cotton. However, blending polyester with other fabrics might compromise its hydrophobicity [20].

Silk is a natural material made by silk moth caterpillars, such as those of the domesticated silk moth, *Bombyx mori*, and of the Robin moth, *Hyalophora cecropia*. These caterpillars produce and use silk for spinning their cocoons [22–24], which are structures that consist of hydrophobic and semi-impermeable membranes [25, 26] that protect the developing moth residing inside from harsh abiotic and biotic conditions [26–28]. Although less frequently used as a resource material for task-specific PPE and face coverings, silk is already used in biomedical applications such as surgical sutures [29], and current research has examined its utility as a biomaterial for many biomedical and human health applications [30, 31]. Moreover, silk possesses certain traits that warrant its potential use, such as its naturally hydrophobic nature and its inherent antimicrobial, antibacterial, and antiviral properties [29, 32–34]. Previous work examining the use of commercially available fabrics for improvised face coverings has also shown that silk possesses some capacity as an antimicrobial barrier when used alone for the fabrication of face coverings [35]. In addition, recent work has found that the filtration efficiency of silk, as tested in aerosol particulate tests, increases significantly with the number of layers [12].

## Methods

### Materials and surgical masks tested

**Fig 1:**
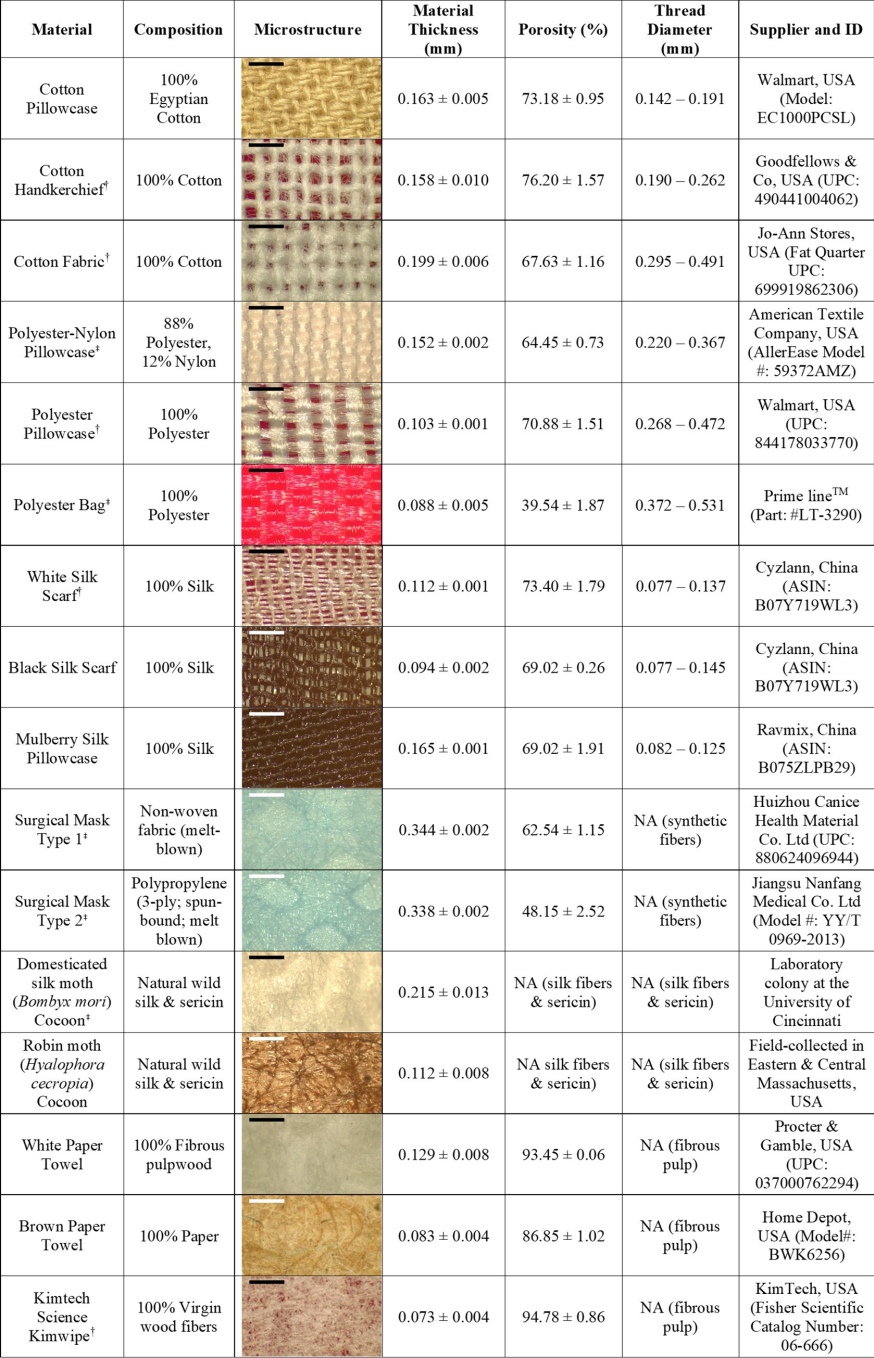
Information on the materials tested in this study. ‡ = Images with adjusted contrast and brightness (+20% brightness/-20% contrast) to emphasize the material’s weave structure;† = Images taken on a red background to accentuate the material’s color. Microstructure images were taken using a stereomicroscope with built-in digital camera (Leica, Model EZ4 W). Table contains information on the composition, microstructure, thickness (mm; mean ± SEM), mass-void determined porosity (%; mean ± SEM), thread diameter (mm; range), and supplier information. Black or white bars on microstructure images are scale bars (= 1.0 mm). ASIN = Amazon Standard Identification Number; UPC = Universal Product Code. Information on the composition of each material is based on how each was marketed. Melt blown materials (e.g., surgical masks) are constructed with polymers (e.g., polypropylene). Each material had three separate swatches tested for porosity (%) and thickness (mm), and thread diameter range was measured from five randomly chosen threads from a single optical image. In the Porosity category, a value of not applicable (NA) was given to materials that cannot logistically be fashioned into face coverings (i.e., cocoons). Similarly, NA values were given to materials that had no actual thread weaves (i.e., only fibers present) precluding measurement, in the thread diameter category.

We tested six material groups for contact angle, saturation propensity, and gas exchange rates. We also examined three of these material groups when these materials were tested as either single or multiple layers in trials measuring droplet penetration resistance, and then compared the performance of sewn masks made from cotton, polyester, or silk materials with commercially available surgical masks in their resistance to aerosolized spray (see Fig 1 for specific information on each material group and surgical mask type).

The material groups consisted of animal-derived silk that was either unmanipulated or processed, processed plant-derived fabric (100% cotton), processed synthetic fabric (polyester), and water-absorbent material as positive controls. These processed fabrics (cotton, polyester, and silk) represent commonly available materials that can be readily used for making protective layers and face coverings. For processed silk, we tested both washed and unwashed silk to examine if the material properties of silk might be altered by standard cleaning techniques (i.e., washing).

For animal-derived silk that was unmanipulated, we took domesticated silk moth (*Bombyx mori*) cocoon samples from our current laboratory colony (3^rd^ generation reared; Department of Biological Sciences, University of Cincinnati) and robin moth (*Hyalophora cecropia*) cocoons collected outdoors from Eastern and Central, Massachusetts between 2013–2016 [26]. For animal-derived processed silk materials, we tested unwashed and washed 100% silk scarves that were either black or white in color, and unwashed and washed 100% mulberry silk pillowcases. Subsets of the silk material were washed with hair shampoo according to instructions outlined by the distributor, in order to create the washed silk group. For processed plant-derived material, we tested a 100% cotton handkerchief, 100% cotton fabric, and a 100% Egyptian cotton pillowcase. The synthetic materials that we tested included a pillowcase that was a blend of 88% polyester– 12% nylon, a 100% polyester pillowcase, and a 100% polyester drawstring bag. Positive controls (i.e., paper towels) consisted of a generic brand of a white paper towel, a brown paper towel, and Kimwipes. Fabrics used for face coverings that were tested in aerosolized spray experiments were made from 100% mulberry silk material, 100% cotton material, and 100% polyester material. Surgical masks tested in our study were purchased from local retail stores.

### Contact angle trials

We compared the different material groups in their level of hydrophobicity, functionally characterized by their ability to block small water droplets, vehicles for the transmission of the virus underlying COVID-19 [36], in contact angle trials [37]. Contact angle trials measure the behavior of a water droplet deposited onto the surface of a material (using the sessile drop technique; see below), and the hydrophobicity of the test material is based on the angle produced by the edge of a water droplet contacting the surface. Greater hydrophobicity was defined as the starting contact angles of droplets being greater than 90°, which produces increased resistance to the penetration of droplets into the material. We assessed hydrophobicity by first measuring the contact angle behavior of an individual small water droplet (5 μL and 2 μL volumes) deposited onto the surface of these materials using the sessile drop technique. In these tests, greater contact angles that are more consistent over time indicate greater hydrophobicity.

Contact angle data for 5 μL and 2 μL water droplet trials were collected using an experimental setup based on those used previously [37]. The droplet volumes were based on the range of values previously used to test natural materials and fabrics [27, 38]. We deposited the water droplet (5 or 2 μL) onto the material piece using a pipette. We avoided any effects of kinetic energy on the contact angle formed by the droplet by ensuring the droplet was in contact with both the pipet tip and the surface of the material piece prior to final deposition [39]. We used a high-resolution digital camera (Micro 4/3 Lumix SLR, Panasonic Corporation) to capture trial images. During all trials, the camera was kept level with the water droplet and test material. We performed trials on a plastic platform that was positioned horizontally and leveled using a leveler (Bullseye Surface Level, Empire Level). For each trial, we obtained three mean contact angle measurements (mean angle of the contact angle of the left and right sides of the droplet as seen in images): the starting contact angle (time = 0 s, the first image that the pipette tip was completely out of frame), the dynamic contact angle (mean contact angle, sampled every five seconds, and averaged at the end of the trial), and the final contact angle (defined as the last reliable image in which the contact angle could be determined or at *t* = 120s, the time at which we terminated trials). We tested the contact angle of 5 μL and 2 μL water droplets separately.

Images were sampled every second for a total duration of two minutes and then uploaded to ImageJ 1.52a [40] for analysis. The two points of contact were then identified as the outer most points at which the droplet touched the material surface. A straight line was then drawn with the angle tool connecting the two points of contact, parallel to the plane of the material, and the angle line was drawn tangential to the point of contact between the droplet and the material. This technique was done for both the right and left side of the droplet and then averaged to obtain the mean contact angle [41]. A contact angle measurement was determined unreliable if either of the two points of contact or the curvature of the droplet could not be determined.

### Saturation propensity trials

We measured the saturation propensity of a water droplet (2 μL), i.e., the absorption of a droplet by the test material, to examine the ability of water to penetrate through the material. Saturation propensity was used to test the permeability of the test material. For each trial, we applied a 2 μL water droplet to the surface of the material using a pipette. The water droplet was applied using a similar technique as in contact angle trials, by ensuring that the droplet was in contact with the material first before depositing the droplet. After depositing the droplet, we waited 1-minute before taking an image of the material to measure the total area that the water droplet had spread within the material. Images were analyzed using ImageJ 1.52a [40]. If the water droplet was not fully absorbed at the end of 1-minute, we measured the area of the water droplet that remained on the surface of the material.

### Gas exchange trials

We measured the rate of gas exchange over a 24-hour period through the different materials, in order to examine the ability of water (vapor) to penetrate through the material. Gas exchange rates are a measure of porosity and therefore breathability [42]. We tested the rate of gas exchange for each material by using methods that were modified from previous studies [43, 44]. We built an airtight holder for material swatches through which only water vapor was allowed to evaporate. The apparatus was created from a 0.3 mL micro reaction vessel with a hole in the rubber seal to keep the vessel airtight. Each micro reaction vessel was filled with water (300 μL), covered with the material swatch and airtight cap, and then placed on an electronic balance in the room to obtain the initial weight and to measure the weight change after a 24-hour period. We recorded the ambient temperature and humidity of the room for the duration of these tests to correct for the water vapor transfer rate [45]. In addition to gas exchange trials, we obtained an additional measure of porosity for the different material types and surgical masks using void mass measurements [44]. In the gas exchange experiment, although cotton can become readily wet by direct contact from small aqueous drops, this was not an issue because the tested materials were not in direct contact with the water in the micro reaction vessels. During the trials, water vapor was capable of passing through the openings in the cotton material, as with the other material types. Gas exchange resulted from the difference in the relative humidity between the inside and the outside of the micro reaction vessel. This gradient is what drives water vapor transfer through the materials during trials.

### Droplet absorption: Single and multilayered silk

**Fig 2:**
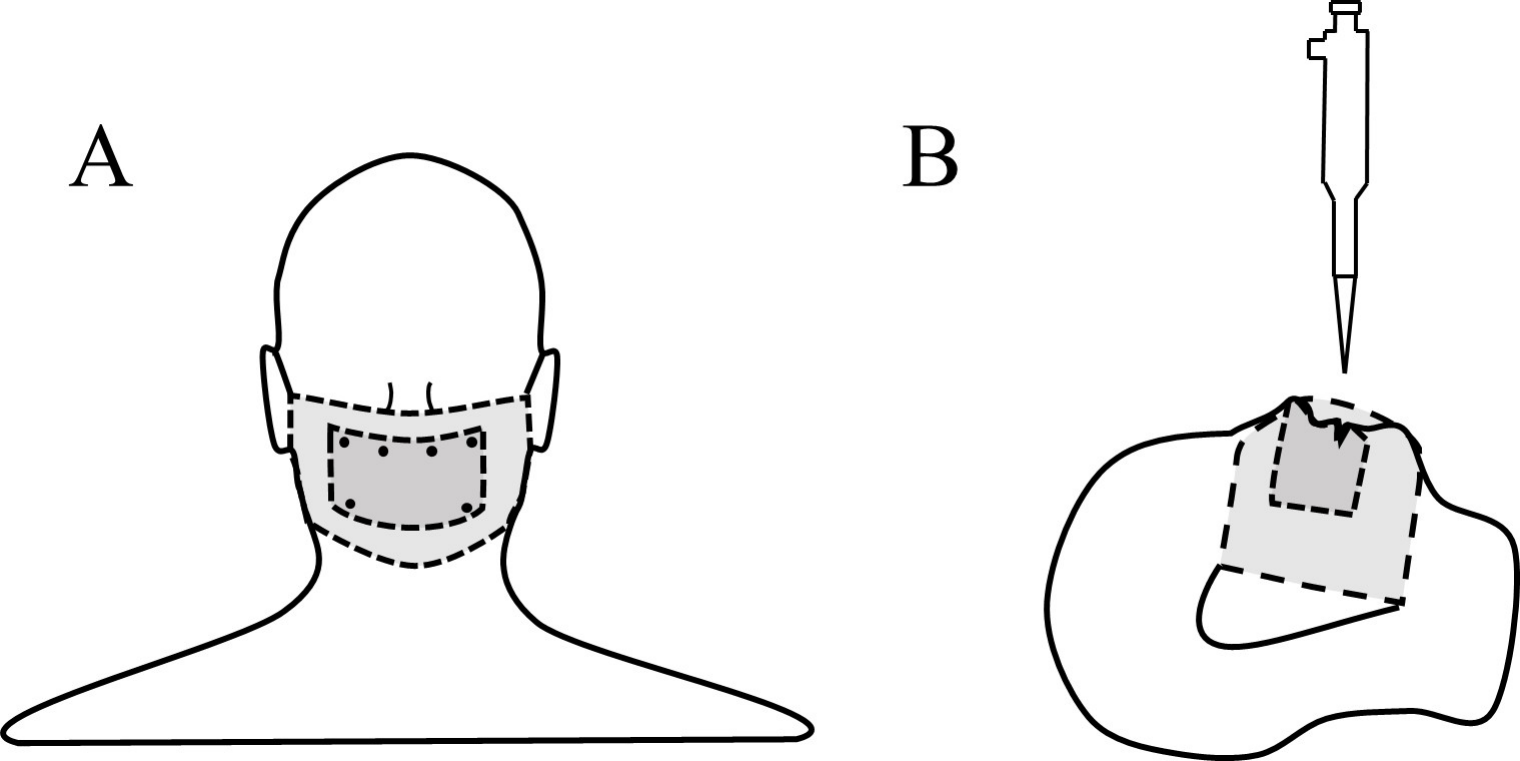
Experimental set-up and index card attachment for droplet tests with different layers of silk. (A) Blank index cards were held to the mannequin head using pins. Silk fabric pieces were placed on top of the index card and held in place on the mannequin head during trials in a way consistent with face coverings. (B) Delivery of the droplet during trials with the mannequin head in a horizontal position. For both (A) and (B), the dark-grey layer on the mannequin head represents the index card placement while the covering denoted by the light-grey layer represents the test fabric pieces.

We determined how increasing the number of layers of silk affects its ability to be an effective barrier. We compared how one, two, or three layers of silk can hamper the penetration of a 2 μL water droplet, when silk was either washed (n = 3) or unwashed (n = 3). For each trial, we placed a 7.62 cm by 12.70 cm blank index card (Walmart Inc., AR, USA) on a Styrofoam mannequin head that covered the nose, mouth, and upper cheek areas (left and right) of the mannequin head’s face. The index card was held in place with pins (Fig 2A). This index card was then in turn covered by the silk fabric pieces used in trials (Fig 2A). During trials, the mannequin head was lying in a horizontal position (Fig 2B). As done with contact angle trials (see above), a pipette was used to apply droplets to the nose (n = 1), mouth (n = 1), and upper cheek (n_left_ = 1; n_right_ = 1) areas for a total of four 2 μL droplets per technical replicate, and three material replicates (i.e., three distinct fabrics from the same group) were tested for silk materials (washed, n = 3, and unwashed, n = 3). Washed and unwashed silk had 3 material replicates with 3 technical replicates per material. Each trial was completed when the 2 μL droplet was no longer present on the surface of the silk, either through absorption or evaporation. After each test, we then placed the index card on a flatbed scanner (Canon MG2220, Canon, Inc.), and created a digital image of the index card. Using ImageJ v1.52a [40], we examined the index cards for any discoloration due to the deposited droplet. Blank index cards were used to identify possible potential discoloration in the card from the manufacturing process that would create a false positive detection during image analysis. These were identified as small dark points on the card that differed from discoloration caused by the droplet.

### Aerosolized water droplet spray tests

We compared the different fabric types (cotton, polyester, and silk) and commercially available surgical masks, in terms of the penetration of aerosolized droplets delivered as spray through the material, via a modified custom apparatus [46]. We also tested the penetration of aerosolized spray after sterilization, where face coverings were sterilized a total of five times using a dry-heat oven at 70°C [47].

The velocity of the spray was determined through the relationship of flow rate and velocity using the following equations for flow rate (m^3^/s):

$$Q=\frac{v}{t}$$ (1)

where *Q* is the flow rate (m^3^/s), *v* is the volume (m^3^), and *t* is time (s). The relationship between flow rate and velocity is as follows:

Q=AV (2)

where *Q* is flow rate (m^3^/s), *A* is the cross-sectional area of the cylinder (m^2^), and *V* is the velocity (m/s). We solved for velocity by first calculating the flow rate (*Q*) from Eq (1) and then rearranging Eq (2). We recorded each spray using a camera (Logitech HD Pro C920) and weighed the apparatus before and after each spray. The aerosolized spray had an average velocity of 0.88 ± 0.04 m/s with each spray containing 0.125 ± 0.05 mL of liquid. Although a real human cough has an extreme amount of variability in droplet size, cough plume, and other characteristics [48], our device based on a similar experimental design [46] represents an extreme case in which a person openly coughs in close proximity without any protective barrier.

### Single- and double-layer fabric barrier aerosolized spray test

**Fig 3:**
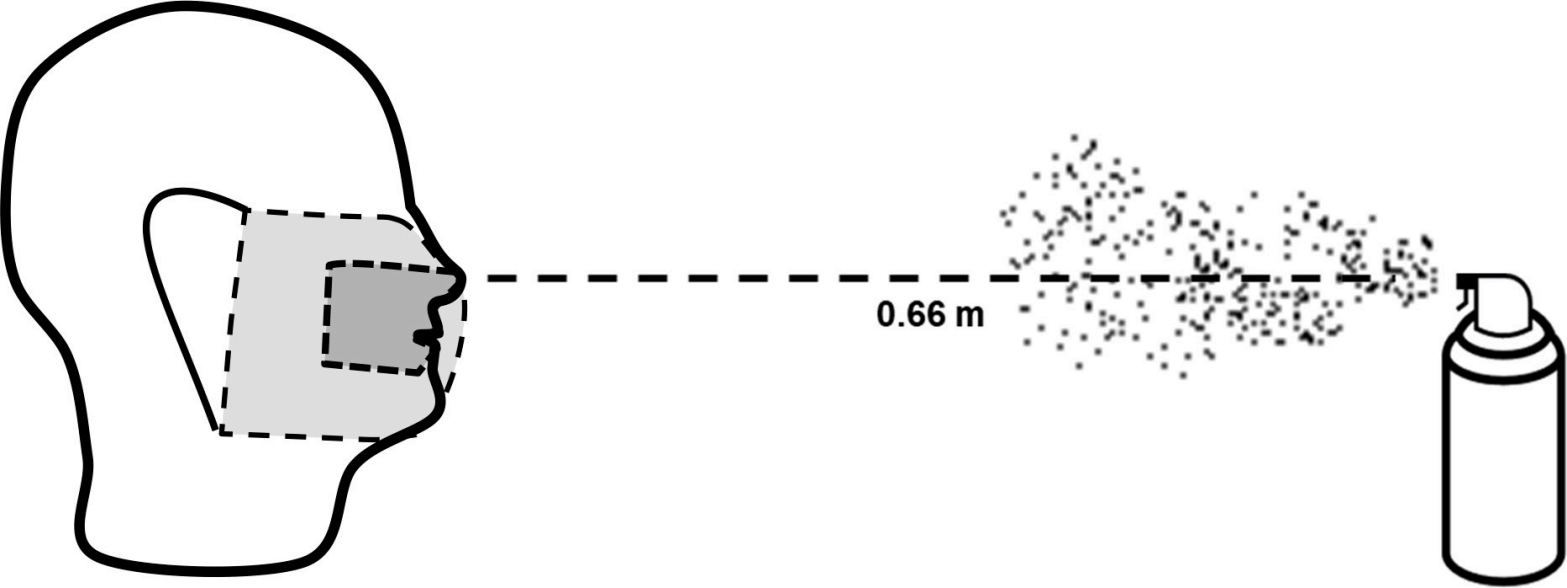
Aerosolized spray experimental set-up with mannequin head (no face covering or surgical mask during trials) and aerosolizing apparatus. Prior to each test, the apparatus was filled to 82 kPa. The dark-grey covering represents the blank index card placement while the light-grey covering represents the fabric barrier, face covering, or surgical mask tested in trials.

We compared 100% cotton, 100% polyester, and 100% silk (washed and unwashed) as either a single layer or double-layered fabric barrier for aerosolized spray tests. Double layers were made by placing two layers of fabric directly on top of each other. To produce a tight fit, the single and double-layered fabrics were attached to the mannequin head with pins. We modified an aerosol can with a standard valve, and added 150 mL of black-dyed water (10 mL black dye, 140 mL water; McCormick, MD, USA). Prior to each trial, the aerosol can was filled to 82 kPa with an air pump and checked using a tire-pressure gauge. For a trial, the Styrofoam mannequin head had either the single or the double-layered barrier positioned directly on a blank index card that was pinned to the face (Fig 3; no ear loops in this test). The mannequin head was then positioned standing upright and placed 0.66 m [48] from the aerosol can (Fig 3). A control group (no fabric barrier on the index card pinned to the mannequin head’s face) was sprayed to provide a baseline of discoloration for comparison. Each trial consisted of a single spraying from the apparatus. The aerosolized droplets were of a random distribution in size with the speed and total volume consistent across trials.

### Face covering and surgical mask aerosolized spray test before and after sterilization

Face coverings were made according to the CDC guidelines for sewn pleated face coverings [7], and were each made with a single material that consisted of either cotton, polyester, or silk. We made three face coverings for each material group (cotton, polyester, silk) and included two brands of disposable surgical masks for comparison in the aerosolized spray test. We used the same aerosol spray apparatus that was used for testing single and double-layered fabric barriers (as above). The face coverings and surgical masks were positioned similarly as the face barriers to cover the index cards. The ear loops of the face coverings and surgical masks were put on the mannequin ears, and further held in place with pins (Fig 3). Initially, these face coverings were tested prior to any sterilization and stretching. After the initial trials, the face coverings were each sterilized using dry heat (70°C) [47] for 1-hour and then retested after a single sterilization and after five sterilizations. After each was sterilized, face coverings were worn for approximately 5-minutes by the same person (A.F.P.) and stretched (i.e., diagonally, horizontally, and vertically) to simulate wear-and-tear. The same face coverings and masks were used across all trials, and for each trial a mask or face covering was only sprayed once per technical replicate. Each material group had three mask or face covering replicates that were each tested three times. After each trial, the index card was scanned to create a digital image of the index card that was then processed in ImageJ 1.52a [40]. The images were converted into 16-bit images to allow grayscale thresholding to isolate and separate pixels darkened by the aerosolizing apparatus. Using a positive control, the threshold value was determined by incrementally increasing the value until both visible spots were sufficiently covered and before there was significant threshold identification on either the white of the card or on the background on which the cards were placed. From this process, we were able to obtain an area and associated identity for every contiguous threshold particle. This tool enabled us to exclude any particles that were obviously not droplets from the aerosolizing apparatus and instead resulted from the experimentation itself. These included (1) holes created by the pins securing the card to the fabric, (2) large shadowed portions of the card created by unintentional bending or creasing of the card during experimentation, and (3) large fabric remnants or other debris found on the card. After these areas were excluded, the total sum area of all the threshold particles was calculated.

### Data analysis

In all of our experiments, we tested three different sources for each material type and performed three technical replicates for each material source. Thickness measurements were made in three separate locations randomly selected on the material and then averaged.

For contact angle trials (both larger 5 μL and smaller 2 μL droplets), we compared the different material types in terms of their starting, dynamic (i.e., change over time), and final contact angles during trials, and the magnitude change in contact angle between the start and final measurements. We analyzed starting and final contact angles, and the magnitude change in contact angle, using a one-way ANCOVA with material thickness as a covariate. Dynamic contact angle data were analyzed using a generalized linear mixed-effect model (GLMM) with *group* and *time* as a fixed effect interaction, and *fabric sample* as the random effect. Individual models were compared against a null using a likelihood ratio test, and the conditional and marginal r^2^ are reported for each model [49]. We analyzed saturation propensity using a one-way ANCOVA with material thickness as a covariate. Gas exchange data were first log_10_-transformed to meet assumptions of normality, and then compared among material types using a one-way ANOVA.

Comparisons of the percentage of samples that were penetrated by a 2 μL water droplet for either single or multilayered silk fabric layers were analyzed using a Fisher’s Exact omnibus test, which was then followed by pairwise Fisher’s Exact tests with Bonferroni correction (α = 0.016). Relative to when no face covering was present over a testing surface, we compared the capability of face coverings (cotton, polyester, and silk) and surgical masks to repel aerosolized droplets (i.e., resist penetration and saturation by aerosol droplets delivered via spray) using a one-way ANOVA. All data were analyzed in R [50]. For all ANCOVA and ANOVA tests, we reported the two effect sizes of eta squared (η^2^) and partial eta squared (η_p_^2^). Significance was set to α = 0.05 except when adjusted for multiple pairwise comparisons.

## Results

### Testing the performance of material for use as protective layers or face coverings

The material groups differed significantly in starting contact angles for both droplet volumes tested (5 μL–ANCOVA: F_6,55_ = 16.88, P<0.001; η^2^ = 0.62, η_p_^2^ = 0.64; 2 μL–ANCOVA: F_6,55_ = 20.36, P<0.001; η^2^ = 0.68, η_p_^2^ = 0.69). In all trials, silk-based materials (*B*. *mori* and *H*. *cecropia* cocoons, unwashed and washed silk) were found to be hydrophobic, as they had mean starting contact angles greater than 90° (Table 1). In contrast, cotton, polyester, and paper towel materials were classified as hydrophilic as the starting angles of cotton and polyester were far below 90° and paper towel had immediate droplet absorption (Table 1). The thickness of materials was significantly related to the starting contact angle for both droplet volumes tested, such that thicker materials, regardless of material type, had larger starting contact angles (5 μL–ANCOVA: F_1,55_ = 4.47, P = 0.039; η^2^ = 0.03, η_p_^2^ = 0.08; 2 μL–ANCOVA: F_1,55_ = 6.87, P<0.05; η^2^ = 0.04, η_p_^2^ = 0.11).

**Table 1:** Contact angle metrics (mean ± SEM).

Material Group	Starting CA (°)	Final CA (°)	Magnitude (°)
5 μL Water Droplet			
*B*. *mori* cocoon	116.96 ± 6.36^a^	94.55 ± 18.86^a^	22.41 ± 20.34^a,b^
*H*. *cecropia* cocoon	92.96 ± 11.10^a,b^	38.69 ± 11.88^b,c,d^	54.26 ± 10.49^a^
100% Silk (Unwashed)	120.09 ± 2.73^a^	69.79 ± 27.62^a,b^	50.30 ± 26.37^a,b^
100% Silk (Washed)	107.60 ± 19.07^a,b^	41.69 ± 24.59^b,c^	65.90 ± 24.82^a^
100% Cotton	42.81 ± 37.21^c,d^	11.56 ± 10.01^c,d^	31.24 ± 27.29^a,b^
Polyester	61.16 ± 26.84^b,c^	30.66 ± 21.83^c,d^	30.50 ± 20.04^a,b^
Paper towel (Positive control)	0.00 ± 0.00^d^	0.00 ± 0.00^d^	0.00 ± 0.00^b^
2 μL Water Droplet			
*B*. *mori* cocoon	102.03 ± 9.48^a^	64.28 ± 17.07^a^	37.75 ± 10.45^a,b^
*H*. *cecropia* cocoon	85.68 ± 14.81^a,b^	36.18 ± 12.12^a,b,c^	49.50 ± 16.96^a^
100% Silk (Unwashed)	120.09 ± 8.66^a^	64.38 ± 25.86^a^	55.71 ± 21.02^a^
100% Silk (Washed)	95.17 ± 18.02^a^	54.07 ± 27.47^a,b^	41.11 ± 22.37^a,b^
100% Cotton	34.17 ± 30.61^c,d^	15.08 ± 13.21^c^	19.09 ± 16.92^a,b^
Polyester	46.98 ± 22.35^b,c^	18.38 ± 9.16^b,c^	28.59 ± 17.71^a,b^
Paper towel (Positive control)	0.00 ± 0.00^d^	0.00 ± 0.00^c^	0.00 ± 0.00^b^

Three metrics of contact angle (CA) including starting contact angle, final contact angle, and the magnitude change from the start to final contact angles for 5 μL and 2μL water droplets (mean ± SEM). All groups had 3 material replicates with 3 technical replicates per material. Material groups that share the similar letter are not significantly different from each other (Tukey HSD post-hoc tests, α = 0.05) in each respective metric and water droplet test.

**Fig 4:**
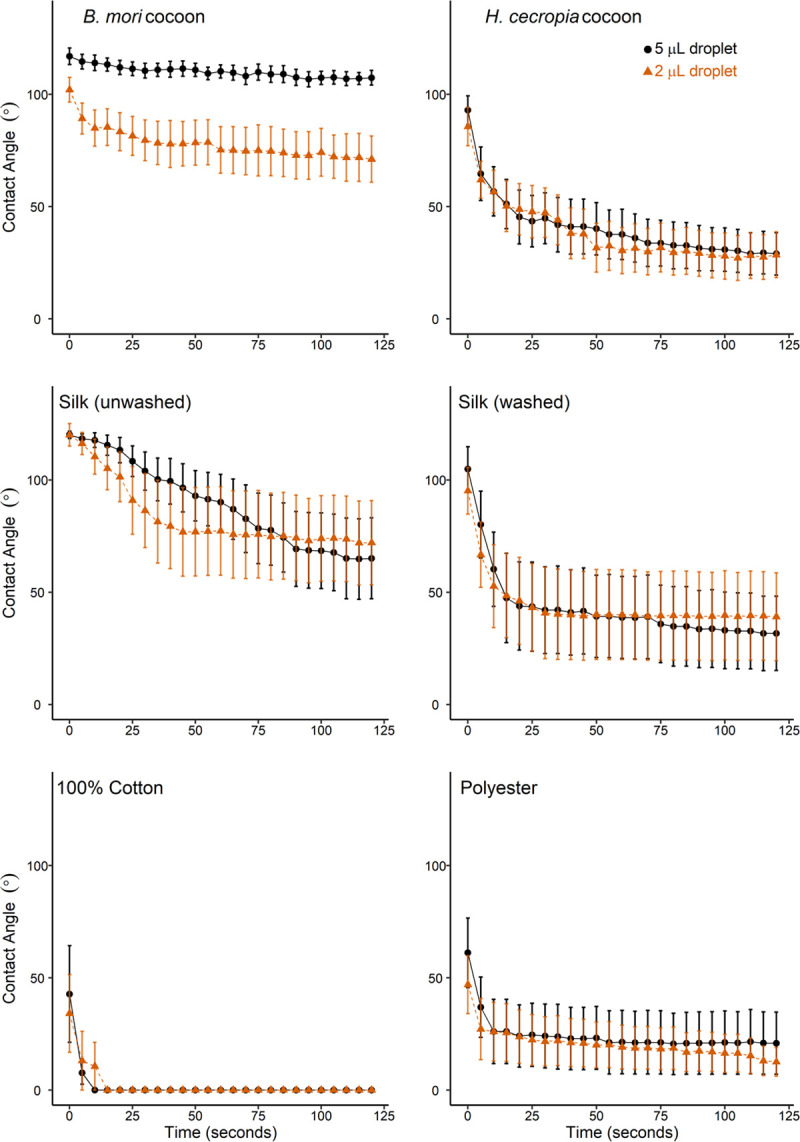
Mean dynamic contact angle (°) of a 5 μL (black) and 2 μL (orange) water droplet for each material group over a 2-minute trial duration. For both 5 μL and 2 μL droplets, *B*. *mori*, *H*. *cecropia*, washed, and unwashed silk all had starting contact angles above 90° which indicated a hydrophobic surface, while the other fabric types (i.e., cotton and polyester) had contact angles less than 90°, indicating a hydrophilic surface. The positive control (paper towel) is not shown because the water droplet was immediately absorbed and therefore no contact angle could be measured in any of the trials.

We found a significant interaction between material group and time for mean dynamic contact angles (GLMM: 5 μL– *χ*^2^ = 778.58, df = 13, P<0.001; marginal r^2^ = 0.62, conditional r^2^ = 0.94; 2 μL– *χ*^2^ = 549.18, df = 13, P<0.001; marginal r^2^ = 0.46, conditional r^2^ = 0.93; Table 2). Hydrophilic materials (cotton, polyester, paper towel), in combination with a lower mean starting contact angle, had a faster change in contact angle during trials, such that the droplet was almost immediately absorbed (Fig 4 and Table 2). In contrast, the droplet placed on hydrophobic materials (all silk-based groups) stayed on longer and was not readily absorbed, resulting in a gradual change over time (Fig 4 and Table 2).

**Table 2:** Summary of mixed-effect models for the dynamic contact angle of 2 μL and 5 μL water droplets.

	2 μL Contact Angle			5 μL Contact Angle		
*Predictors*	*Estimates*	*CI*	*P*	*Estimates*	*CI*	*P*
(Intercept)	87.72	66.49 – 108.96	<0.001	114.13	95.10 – 133.16	<0.001
Time	-0.16	-0.20 – -0.12	<0.001	-0.07	-0.11 – -0.02	0.004
*H*. *cecropia*	-29.88	-59.91 – 0.15	0.051	-54.74	-81.66 – -27.82	<0.001
100% Cotton	-79.31	-109.34 – -49.28	<0.001	-106.58	-133.50 – -79.66	<0.001
Paper Towel	-87.72	-117.76 – -57.69	<0.001	-114.13	-141.05 – -87.21	<0.001
100% Silk (unwashed)	16.02	-14.01 – 46.06	0.296	6.24	-20.68 – 33.16	0.649
100% Silk (washed)	-31.64	-61.67 – -1.61	0.039	-51.63	-78.55 – -24.71	<0.001
Polyester (synthetic)	-58.49	-88.52 – -28.45	<0.001	-81.81	-108.73 – -54.89	<0.001
Time * *H*. *cecropia*	-0.16	-0.23 – -0.10	<0.001	-0.24	-0.31 – -0.18	<0.001
Time * 100% Cotton	0.06	-0.00 – 0.12	0.069	-0.03	-0.09 – 0.04	0.443
Time * Paper Towel	0.16	0.10 – 0.22	<0.001	0.07	0.00 – 0.13	0.043
Time * 100% Silk (unwashed)	-0.18	-0.24 – -0.11	<0.001	-0.45	-0.51 – -0.38	<0.001
Time * 100% Silk (washed)	-0.04	-0.10 – 0.03	0.248	-0.26	-0.32 – -0.19	<0.001
Time * Polyester	0.02	-0.05 – 0.08	0.591	-0.06	-0.13 – 0.00	0.052
Marginal R^2^ / Conditional R^2^	0.463 / 0.932			0.619 / 0.938		

The asterisk indicates an interaction term in the GLMM between *time* and the pertinent material group.

Final contact angles also differed significantly between groups for both droplet volumes tested (5 μL–ANCOVA: F_6,55_ = 13.02, P<0.001; η^2^ = 0.62, η_p_^2^ = 0.64; 2 μL–ANCOVA: F_6,55_ = 8.72, P<0.001; η^2^ = 0.52, η_p_^2^ = 0.56). Overall, the pattern of final contact angles for both droplet volumes showed that unprocessed (*B*. *mori* and *H*. *cecropia* cocoons) and processed silk (washed and unwashed 100% silk) had the greatest final contact angles (Table 1). Polyester had intermediate final contact angles of the materials tested (Table 1). 100% cotton and paper towel materials had the smallest final contact angles of all material groups (Table 1). Thickness was significantly related to the final contact angle for all droplet trials within each material group (5 μL–ANCOVA: F_1,55_ = 25.04, P<0.001; η^2^ = 0.16, η_p_^2^ = 0.31; 2 μL–ANCOVA: F_1,55_ = 19.43, P<0.001; η^2^ = 0.15, η_p_^2^ = 0.26; Table 1), where final contact angles, within all material types, were larger with increasing thickness.

The magnitude of change from the starting to final contact angles was significantly different across material groups for both droplet volumes tested (5 μL–ANOVA: F_6,56_ = 3.48, P<0.01; η^2^ = 0.27; 2 μL–ANOVA: F_6,56_ = 3.93, P<0.01; η^2^ = 0.30; Table 1). There was a larger change in contact angle for hydrophobic materials due to the larger initial starting contact angle relative to that of hydrophilic materials. Post hoc pairwise comparisons, however, indicated only significant differences between the paper towel control group and each of the material groups (Table 1).

The saturation propensity of a 2 μL water droplet significantly differed between material groups (ANCOVA: F_6,49_ = 55.875, P<0.001; η^2^ = 0.74, η_p_^2^ = 0.87), with cotton and paper towel having the largest droplet spread area followed by the remaining groups (Table 3). Thickness was significantly related to droplet spread area (ANCOVA: F_1,49_ = 7.14.884, P<0.001; η^2^ = 0.03, η_p_^2^ = 0.23), as droplet spread area increased with thickness. However, there was a significant interaction between thickness and fabric type (ANCOVA: F_6,49_ = 9.772, P<0.001; η^2^ = 0.13, η_p_^2^ = 0.54). The significant interaction between thickness and material group indicates that the effect of thickness on droplet spread varies for the different material types, highlighting the complexity of interactions between material type and thickness. For instance, droplet spread increased on polyester material as its thickness increased, demonstrating that saturation occurred as the material absorbed the droplet via spreading across the fabric. As the droplet spread area was not as great as cotton or paper towel, further saturation through the fabric was mitigated by increased thickness, with water not penetrating any deeper into the polyester fabric. In contrast, cotton and paper towel materials had the largest saturation area, yet had decreased droplet spread with increasing thickness. This indicates that these materials readily absorbed the droplet, but water saturated the fabric by directly and quickly penetrating through the material. Increased thickness in cotton or paper towel did not prevent these materials from getting saturated. This further demonstrates that cotton is hydrophilic, since it readily absorbs droplets as did the paper towel positive controls. Overall, the droplet spread for silk (unprocessed and processed) remained constant as the thickness of silk material increased. Gas exchange, a proxy for breathability, significantly differed between groups (ANOVA: F_6,56_ = 16.643, P<0.001, η^2^ = 0.64). *B*. *mori* cocoons and cotton material had the highest mean gas exchange rates relative to the other groups (Table 3).

**Table 3:** Saturation (mm^2^) from a 2 μL droplet for material groups where absorption was present (100% cotton, positive control, unwashed silk, synthetic polyester) and not present (*B*. *mori*, *H*. *cecropia*, washed silk) after 60 seconds, and gas exchange rates after a 24-hour period.

Material Group	Saturation Area (mm^2^) ± SEM	Permeability (g/m ∙ s ∙Pa) ± SEM
*B*. *mori* cocoon	1.59 ± 0.12^c^	1.92^−09^ ± 1.81^−10, a^
*H*. *cecropia* cocoon	4.98 ± 1.10^c^	7.03^−10^ ± 7.58^−11 c,d^
100% Silk (Unwashed)	11.96 ± 7.23^b,c^	8.74^−10^ ± 1.03^−11 c,d^
100% Silk (Washed)	5.06 ± 1.47^c^	9.45^−10^ ± 1.45−11 c,d
100% Cotton	86.52 ± 17.67^a^	1.35^−09^ ± 6.85^−11 a,b^
Polyester	26.26 ± 10.94^b^	8.44^−10^ ± 1.06^−11 b,c^
Paper Towel (Positive Control)	69.69 ± 22.82^a^	7.00^−10^ ± 2.58^−10 d^

There were significant differences in saturation area between the material groups. All groups had 3 material replicates with 3 technical replicates per material. For gas exchange rates, silk is as porous as synthetic materials and less porous than 100% cotton. A one-way ANOVA test indicated a significant difference between material groups. Groups that share the same letters are not statistically different from each other (Tukey HSD post-hoc tests, α = 0.05).

To examine how multiple layers of silk affect the penetration of droplets, we compared the ability of a 2 μL water droplet to penetrate single and multiple fabric layers. We found that the droplet penetration of silk fabric significantly decreased as the layers of silk increased from a single layer (47%, n = 72 droplets), to either double (3%, n = 72 droplets) or triple (1%, n = 72 droplets) layers (Fisher’s Exact, P<0.001), but two and three layers of silk did not differ from each other.

### Testing single- and double- Layer fabric barriers to aerosolized spray

As the public typically wears improvised face coverings that may have one or two layers, we compared the capability of single and double layer fabric barriers made out of either silk (washed and unwashed), cotton, or polyester fabrics to resist penetration by aerosolized droplets in spray tests. We found that each of the fabric groups significantly prevented droplet penetration relative to the control condition of no fabric barrier, when fabric barriers had one-layer (ANOVA: F_5,42_ = 18.66, P<0.001, η^2^ = 0.69; P<0.05 for all post-hoc comparisons between the different fabric groups and the control) or two-layers (ANOVA: F_5,42_ = 29.50, P<0.001; η^2^ = 0.78). However, there were no differences in the ability to prevent aerosol droplet penetration between the different fabric groups (P>0.05 for all Tukey HSD post-hoc comparisons) when the fabric barriers had one or two layers.

### Exposure of face coverings and surgical masks to aerosolized spray before and after sterilization

To examine the effects of sterilization, we compared face coverings made from our different test fabrics using recommendations from the CDC [7], with surgical masks. Discoloration of the test surface from the aerosolized spray remained the same for all tested groups with no sterilization (ANOVA: F_4,49_ = 0.99, P = 0.42), one sterilization (F_4,49_ = 0.98, p = 0.43), and five sterilizations (F_4,49_ = 1.702, P = 0.17). This occurred despite significant differences in the thickness of the different face coverings and surgical masks (ANOVA: F_3,41_ = 713, P<0.001; η^2^ = 0.98). Cotton face coverings were the thickest (0.367 ± 0.004 mm, n = 3), followed by masks (0.341 ± 0.008, n = 3), silk (0.306 ± 0.005 mm, n = 3) and then polyester (0.216 ± 0.008 mm, n = 3).

## Discussion

Protective layers and face coverings made from 100% silk, a naturally produced commonly available material, are hydrophobic and can effectively impede the penetration and absorption of both liquid and aerosolized water droplets. The hydrophobicity of silk fabric is further enhanced when used in multiple layers, which when combined, are still thinner than most cotton materials and standard PPE such as surgical masks. Our results demonstrate that the greater hydrophobicity of silk relative to other fabrics, such as cotton and polyester, can make it more effective at impeding droplets, which is a common transmission pathway for the virus that underlies COVID-19 [36].

Silk performs similarly to surgical masks when layered over respirators, as they would occur in clinical settings, yet has the added advantage of having the ability to be easily cleaned through washing for multiple use. Recent work has also aimed at making synthetic, reusable hydrophobic layers to layer on top of respirators [51]. The use of natural silk material to protect PPE adds to these initiatives, but with the added benefits of silk’s inherent beneficial properties and accessibility of silk for both commercial and public use. Here, the sericulture, textile, and garment industries, along with their supply networks and infrastructure, potentially have a direct pathway to becoming important partners against the current COVID-19 pandemic and in future public health emergencies in which PPE may again be in short reserve.

A limitation of respirators and masks, but especially of any face coverings, is that normal breathing can be hampered when worn, and this difficulty increases with thickness. Prolonged use also exposes individuals to added risks, as they increase the local humidity around the area upon which it is worn (>90% relative humidity) [52], thereby creating a potential pathway for the virus to travel due to the trapped moisture near the face (e.g., close proximity to the mouth and nose) that inadvertently increases wetness [5, 53]. Increased humidity underneath these items, exacerbated when worn in hot and humid environments, significantly decreases their wearability because of higher friction and skin moisture [54]. This creates discomfort and can result in an individual unintentionally touching their face or their removal of the face covering completely. In addition, under normal wearing conditions, when water comes in contact with cotton and polyester, these materials readily absorb water and can become saturated, due to their hydrophilic nature. When this occurs, these materials can become thicker, due to the absorption and retention of water (direct), and the absorption of vapor (indirect). This increased thickness can therefore hamper gas exchange. Our results suggest that these limitations and their accompanying risks can be mitigated by silk, at least when it is used in the fabrication of face coverings.

Currently, public health recommendations focus on cotton material for face coverings [17]. We found that cotton materials are hydrophilic, and readily allow droplets to rapidly penetrate and saturate the fabric like a sponge. Therefore, face coverings made out of these materials may quickly become reservoirs of virus and act as conduits for viral transmission when worn, even after a short time [5, 6, 30]. Face coverings made out of polyester face these same limitations, as we found it to be hydrophilic like cotton. Therefore, cloth and polyester face coverings appear to be more suitable for brief, one-time use. In contrast, silk’s hydrophobicity and lack of capillary action [26], can make it a more advantageous material for face coverings that are also thin, light, and breathable. Recent recommendations by the World Health Organization have also mentioned combining hydrophilic and hydrophobic layers when creating face coverings [55], and our work supports the use of silk as a better hydrophobic layer for face coverings that is more effective than either cotton or polyester material that are hydrophilic.

Furthermore, our results also suggest that using multiple layers for silk face coverings can further increase silk’s ability to prevent droplet penetration, thereby enhancing silk’s advantageous hydrophobic properties that can preclude it from becoming a reservoir and conduit for the virus. In addition to the enhanced hydrophobicity of layered silk, layering silk can also significantly increase its filtration efficiency for use as a fabric for face coverings [12]. Recent work testing the aerosol filtration efficiency of common fabrics frequently used for face coverings [12] found that the filtration efficiency of silk increases with the number of layers and that this likely results from silk’s ability to filter aerosols via an electrostatic effect. For example, Konda et al. [12] found that four layers of silk have an 86 ± 5% filtration efficiency for particles <300 nm and an 88 ± 1% filtration efficiency for particles >300 nm (flow rate: 1.2 cubic feet per minute). Taken together, our results and those of previous researchers [12] that have examined the properties of commonly available fabrics, suggest that silk can be a functional resource for fabricating face coverings, in particular those constructed with multiple layers. Silk face coverings can first reduce the penetration and absorption of small droplets, thus reducing saturation (hydrophobicity–our study), and then provide some filtration against aerosol particles (filtration efficiency–[12]), while remaining thin, breathable, and comfortable when worn (our study).

Although our study demonstrates that silk possesses traits that can make it an advantageous material for use as a reusable protective layer for N95 respirators, a limitation of this aspect of our work is that our results were obtained under controlled laboratory conditions. As a protective layer for respirators, our work can now serve as an important springboard for clinical trials that test the efficacy of silk and how its use can benefit workers in health care settings for protecting their PPE. In addition, despite our study showing that silk can be a beneficial material for the construction of face coverings, task-specific N95 respirators are still the most effective and appropriate form of protection against viral transmission. Despite their ability to reduce viral transmission between people when worn [9, 10], we stress that face coverings such as those made with silk form only one part of the necessary armament against viral transmission. For the general public, face coverings are best used in tandem with proper and frequent hand washing, along with strict adherence to recommended social and physical distancing protocols, in order to prevent viral transmission.

In summary, we suggest that silk has untapped potential for use during the current shortage of PPE in the ongoing COVID-19 pandemic and for future health emergencies. Our laboratory-based study highlights the practicality of using current commercially available 100% silk material as a resource for producing protective coverings that can extend the lifetime of N95 respirators, and as a fabric for fashioning face coverings for the general public. Moreover, silk may play a major role in the development of next generation PPE, such as respirator inserts, which can capitalize on its many benefits. For example, silk possesses antimicrobial, antiviral, and antibacterial properties [29, 33], potentially due to the presence of copper, a compound that has antiviral properties and which animals naturally incorporate into their silk [32]. Other fabrics and non-specialized PPE require copper particles to be infused during the manufacturing process [56], an expensive process that could be circumvented by using natural silk fibers. In short, the ability of our society to effectively combat the current COVID-19 pandemic and future public health crises should involve the incorporation of silk material in the development of the next generation of PPE.

## References

[1] Lindsley WG, Martin SB Jr, Thewlis RE, Sarkisian K, Nwoko JO, Mead KR, et al Effects of ultraviolet germicidal irradiation (UVGI) on N95 respirator filtration performance and structural integrity. J Occup Environ Hyg 2015;12:509–17. 10.1080/15459624.2015.1018518 25806411 PMC4699414

[2] Roberge RJ. Effect of surgical masks worn concurrently over N95 filtering facepiece respirators: extended service life versus increased user burden. J Public Health Manag Pract 2008;14:E19–26. 10.1097/01.PHH.0000311904.41691.fd 18287908

[3] Sinkule EJ, Powell JB, Goss FL. Evaluation of N95 respirator use with a surgical mask cover: effects on breathing resistance and inhaled carbon dioxide. Ann Occup 2013;57:384–98. 10.1093/annhyg/mes068 23108786

[4] Ippolito M, Vitale F, Accurso G, Iozzo P, Gregoretti C, Giarratano A, et al Medical masks and Respirators for the Protection of Healthcare Workers from SARS-CoV-2 and other viruses. Pulmonology 2020;26:204–212. 10.1016/j.pulmoe.2020.04.009 PMC7184017 32362505

[5] Li Y, Wong T, Chung AJ, Guo YP, Hu JY, Guan YT, et al In vivo protective performance of N95 respirator and surgical facemask. Am J Ind Med. 2006;49:1056–65. 10.1002/ajim.20395 17096360

[6] Li Y, Guo YP, Wong KC, Chung WY, Gohel MD, Leung HM. Transmission of communicable respiratory infections and facemasks. J Multidiscip Healthc 2008;1:17–27. 10.2147/jmdh.s3019 21197329 PMC3004550

[7] Adams J. Recommendation regarding the use of cloth face coverings, especially in areas of significant community-based transmission. Centers for Disease Control and Prevention. Atlanta, GA: Centers for Disease Control and Prevention, 4 2020 (https://www.cdc.gov/coronavirus/2019-ncov/prevent-getting-sick/cloth-face-cover.html)

[8] Kaltenboeck A, Rajkumar SV. The Case for Masks: Health Care Workers Can Benefit Too. Mayo Clin Proc 2020 95;6:1132–1134. 10.1016/j.mayocp.2020.04.014 32414551 PMC7167549

[9] Leung NH, Chu DK, Shiu EY, Chan KH, McDevitt JJ, Hau BJ, et al Respiratory virus shedding in exhaled breath and efficacy of face masks. Nat Med 2020;26:1–5. 10.1038/s41591-019-0740-8 32371934 PMC8238571

[10] Verma S, Dhanak M, Frankenfield J. Visualizing the effectiveness of face masks in obstructing respiratory jets. Phys. Fluids 2020;32:p.061708. 10.1063/5.0016018 PMC7327717 32624649

[11] Dato VM, Hostler D, Hahn ME. Simple Respiratory Mask. Emerg Infect Dis 2006;12:1033–1034. 10.3201/eid1206.051468 16752475 PMC3373043

[12] Konda A, Prakash A, Moss GA, Schmoldt M, Grant GD, Guha S. Aerosol filtration efficiency of common fabrics used in respiratory cloth masks. ACS Nano. 2020;14:6339–47. 10.1021/acsnano.0c03252 32329337

[13] Centers for Disease Control and Prevention. Understanding the difference. Available at: https://www.cdc.gov/niosh/npptl/pdfs/UnderstandDifferenceInfographic-508.pdf. Retrieved August 3, 2020.

[14] Bauchner H., Fontanarosa P.B. and Livingston E.H., 2020. Conserving supply of personal protective equipment—a call for ideas. Jama 2020;323:1911–1911. 10.1001/jama.2020.4770 32196543

[15] Cooper DW, Hinds WC, Price JM, Weker R, Yee HS. Common materials for emergency respiratory protection: Leakage tests with a manikin. Am Ind Hyg Assoc J 1983;44:720–6. 10.1080/15298668391405634 6650392

[16] Rengasamy S, Eimer B, Shaffer RE. Simple respiratory protection—evaluation of the filtration performance of cloth masks and common fabric materials against 20–1000 nm size particles. Ann Occup Hyg 2010;54:789–98. 10.1093/annhyg/meq044 20584862 PMC7314261

[17] Davies A, Thompson KA, Giri K, Kafatos G, Walker J, Bennett A. Testing the efficacy of homemade masks: would they protect in an influenza pandemic? Disaster Med Public 201;7:413–8. 10.1017/dmp.2013.43 PMC7108646 24229526

[18] Shakya KM, Noyes A, Kallin R, Peltier RE. Evaluating the efficacy of cloth facemasks in reducing particulate matter exposure. J Expo Sci Env Epid 2017;27:352–7. 10.1038/jes.2016.42 27531371

[19] Strasser BJ, Schlich T. A history of the medical mask and the rise of throwaway culture. Lancet 2020;6736:19–20. 10.1016/S0140-6736(20)31207-1 PMC7255306 32450110

[20] Madan GL, Dave AM, Das TK, Sarma, TS. Hydrophilicity of textile fibers. Text Res J 1978;48:662–663.

[21] Sharabaty T, Biguenet F, Viallier P. Investigation on moisture transport through polyester/cotton fabrics. Indian J Fibre Text 2008;33:419–425.

[22] Van Der Kloot WG, Williams CM. Cocoon construction by the Cecropia silkworm I. The role of the external environment. Behaviour. 1953a;5:141–56.

[23] Van Der Kloot WG, Williams CM. Cocoon construction by the Cecropia silkworm II. The role of the internal environment. Behaviour. 1953b;5:157–74.

[24] Chen F, Porter D, Vollrath F. Structure and physical properties of silkworm cocoons. J R Soc Interface 2012;9:2299–308. 10.1098/rsif.2011.0887 22552916 PMC3405738

[25] Kundu J, Dewan M, Ghoshal S, Kundu SC. Mulberry non-engineered silk gland protein vis-a-vis silk cocoon protein engineered by silkworms as biomaterial matrices. J Mater Sci Mater Med 2008;19:2679–89. 10.1007/s10856-008-3398-1 18283532

[26] Guerra PA, Reppert SM. Dimorphic cocoons of the cecropia moth (*Hyalophora cecropia*): Morphological, behavioral, and biophysical differences. PloS one. 2017;12: e0174023 10.1371/journal.pone.0174023 28329006 PMC5362091

[27] Danks HV. The roles of insect cocoons in cold conditions. Eur J Entomol 2004;101:433–8.

[28] Guerra PA, Lawson LP, Gatto LJ, Albright ME, Smith SJ. Architectural evolution in cocoons spun by Hyalophora (Lepidoptera; Saturniidae) silk moth species. Sci Rep 2020;10: 5615 10.1038/s41598-020-62547-1 32221410 PMC7101368

[29] Vepari C, Kaplan DL. Silk as a biomaterial. Prog Polym Sci. 2007;32:991–1007. 10.1016/j.progpolymsci.2007.05.013 19543442 PMC2699289

[30] Nilebäck L, Chouhan D, Jansson R, Widhe M, Mandal BB, Hedhammar M. Silk–silk interactions between silkworm fibroin and recombinant spider silk fusion proteins enable the construction of bioactive materials. ACS Appl Mater Inter 2017;9:31634–44. 10.1021/acsami.7b10874 28846369

[31] Zhou L, Chen X, Shao Z, Zhou P, Knight DP, Vollrath F. Copper in the silk formation process of Bombyx mori silkworm. FEBS lett 2003;554:337–41. 10.1016/s0014-5793(03)01184-0 14623090

[32] Singh CP, Vaishna RL, Kakkar A, Arunkumar KP, Nagaraju J. Characterization of antiviral and antibacterial activity of *Bombyx mori* seroin proteins. Cell Microbiol 2014;16:1354–65. 10.1111/cmi.12294 24628957

[33] Dong Z, Song Q, Zhang Y, Chen S, Zhang X, Zhao P, et al Structure, evolution, and expression of antimicrobial silk proteins, seroins in Lepidoptera. Insect Biochem Mol Biol 2016;75:24–31. 10.1016/j.ibmb.2016.05.005 27180727

[34] Kunz RI, Brancalhão RM, Ribeiro LD, Natali MR. Silkworm sericin: properties and biomedical applications. BioMed Res Int 2016;2016:8175701 10.1155/2016/8175701 27965981 PMC5124675

[35] Wang C, Wu S, Jian M, Xie J, Xu L, Yang X, et al Silk nanofibers as high efficient and lightweight air filter. Nano Res 2016;9:2590–7.

[36] Asadi S, Bouvier N, Wexler AS, Ristenpart WD. The coronavirus pandemic and aerosols: Does COVID-19 transmit via expiratory particles? Aerosol Sci Tech 2020;54:635–638. 10.1080/02786826.2020.1749229 PMC7157964 32308568

[37] Lamour G, Hamraoui A, Buvailo A, Xing Y, Keuleyan S, Prakash V, et al Contact angle measurements using a simplified experimental setup. J Chem Edu. 2010;87:1403–7.

[38] Sun M, Chen Y, Zheng Y, Zhen M, Shu C, Dai Z, et al Wettability gradient on the elytra in the aquatic beetle *Cybister chinensis* and its role in angular position of the beetle at water-air interface. Acta Biomater. 2017;51:408–17. 10.1016/j.actbio.2017.01.022 28069503

[39] Yuan Y, Lee TR. Contact angle and wetting properties In: Surface science techniques Springer, Berlin, Heidelberg, 2013:3–34.

[40] Schneider CA, Rasband WS, Eliceiri KW.NIH Image to ImageJ: 25 years of image analysis. Nat Methods 2012;9:671–675. 10.1038/nmeth.2089 22930834 PMC5554542

[41] Papierowska E, Szporak-Wasilewska S, Szewińska J, Szatyłowicz J, Debaene G, Utratna M. Contact angle measurements and water drop behavior on leaf surface for several deciduous shrub and tree species from a temperate zone. Trees. 2018;32:1253–66.

[42] Tehrani-Bagha AR. Waterproof breathable layers–a review. Adv Colloid Interface Sci 2019;268:114–135. 10.1016/j.cis.2019.03.006 31022590

[43] Rhim JW, Lee JH, Hong SI. Water resistance and mechanical properties of biopolymer (alginate and soy protein) coated paperboards. LWT 2006;39:806–813.

[44] Horrocks NP, Vollrath F, Dicko C. The silkmoth cocoon as humidity trap and waterproof barrier. Comp. Biochem Physiol A Mol Integr Physiol. 2013;164:645–52 10.1016/j.cbpa.2013.01.023 23388210

[45] Gennadios A, Weller CL, Gooding C.H. Measurement errors in water vapor permeability of highly permeable, hydrophilic edible films. J Food Eng. 1994;21:395–409.

[46] Lindsley WG, Reynolds JS, Szalajda JV, Noti JD, Beezhold DH. A cough aerosol simulator for the study of disease transmission by human cough-generated aerosols. Aerosol Sci Tech 2013;47:937–44. 10.1080/02786826.2013.803019 PMC4615563 26500387

[47] Fischer RJ, Morris DH, van Doremalen N, Sarchette S, Matson MJ, Bushmaker T, et al Effectiveness of N95 Respirator Decontamination and Reuse against SARS-CoV-2 Virus. Emerg Infect Dis 2020:26; 10.3201/eid2609.201524 . 32491983 PMC7454118

[48] Tang JW, Nicolle AD, Klettner CA, Pantelic J, Wang L, Suhaimi AB, et al Airflow dynamics of human jets: sneezing and breathing-potential sources of infectious aerosols. PLoS One. 2013;8: e59970 10.1371/journal.pone.0059970 23560060 PMC3613375

[49] Sotirchos ES, Fitzgerald KC, Crainiceanu CM. Reporting of R^2^ statistics for mixed-effects regression Models. JAMA neurology. 2019;76:507. 10.1001/jamaneurol.2018.4720 30715077

[50] R Core Team (2019). R: A language and environment for statistical computing. R Foundation for Statistical Computing, Vienna, Austria URL https://www.R-project.org/.

[51] El-Atab N, Qaiser N, Badghaish HS, Shaikh SF, Hussain MM. A Flexible Nanoporous Template for the Design and Development of Reusable Anti-COVID-19 Hydrophobic Face Masks. ACS Nano. 2020;14:7659–7665. 10.1021/acsnano.0c03976 32432461 PMC7243426

[52] Roberge RJ, Kim JH, Coca A. Protective facemask impact on human thermoregulation: an overview. Ann Occup Hyg 2012;56:102–12. 10.1093/annhyg/mer069 21917820

[53] Gwosdow AR, Stevens JC, Berglund LG, Stolwijk JA. Skin friction and fabric sensations in neutral and warm environments. Text Res J 1986;56:574–80.

[54] Nielsen R, Gwosdow AR, Berglund LG, DuBois AB. The effect of temperature and humidity levels in a protective mask on user acceptability during exercise Am Ind Hyg Assoc J 1987 7 1;48:639–45. 10.1080/15298668791385336 3618476

[55] World Health Organization. Advice on the use of masks in the context of COVID-19: interim guidance, 5 June 2020. World Health Organization; 2020.

[56] Borkow G, Zhou SS, Page T, Gabbay J. A novel anti-influenza copper oxide containing respiratory face mask. PLoS One. 2010;5:e11295 10.1371/journal.pone.0011295 20592763 PMC2892464

